# EMG-based facial gesture recognition through versatile elliptic basis function neural network

**DOI:** 10.1186/1475-925X-12-73

**Published:** 2013-07-17

**Authors:** Mahyar Hamedi, Sh-Hussain Salleh, Mehdi Astaraki, Alias Mohd Noor

**Affiliations:** 1 Faculty of Bioscience and Medical Engineering, Universiti Teknologi Malaysia, Skudai, Johor 81310, Malaysia; 2 Centre for Biomedical Engineering, Transportation Research Alliance, Universiti Teknologi Malaysia, Skudai, Johor 81310, Malaysia; 3 Department of Biomedical Engineering, Science and Research Branch, Islamic Azad University Tehran, Tehran, Iran

**Keywords:** Facial neural activity, Electromyogram, Facial gesture recognition, Feature extraction, Versatile elliptic basis function neural network, Human machine interface

## Abstract

**Background:**

Recently, the recognition of different facial gestures using facial neuromuscular activities has been proposed for human machine interfacing applications. Facial electromyograms (EMGs) analysis is a complicated field in biomedical signal processing where accuracy and low computational cost are significant concerns. In this paper, a very fast versatile elliptic basis function neural network (VEBFNN) was proposed to classify different facial gestures. The effectiveness of different facial EMG time-domain features was also explored to introduce the most discriminating.

**Methods:**

In this study, EMGs of ten facial gestures were recorded from ten subjects using three pairs of surface electrodes in a bi-polar configuration. The signals were filtered and segmented into distinct portions prior to feature extraction. Ten different time-domain features, namely, Integrated EMG, Mean Absolute Value, Mean Absolute Value Slope, Maximum Peak Value, Root Mean Square, Simple Square Integral, Variance, Mean Value, Wave Length, and Sign Slope Changes were extracted from the EMGs. The statistical relationships between these features were investigated by Mutual Information measure. Then, the feature combinations including two to ten single features were formed based on the feature rankings appointed by Minimum-Redundancy-Maximum-Relevance (MRMR) and Recognition Accuracy (RA) criteria. In the last step, VEBFNN was employed to classify the facial gestures. The effectiveness of single features as well as the feature sets on the system performance was examined by considering the two major metrics, recognition accuracy and training time. Finally, the proposed classifier was assessed and compared with conventional methods support vector machines and multilayer perceptron neural network.

**Results:**

The average classification results showed that the best performance for recognizing facial gestures among all single/multi-features was achieved by Maximum Peak Value with 87.1% accuracy. Moreover, the results proved a very fast procedure since the training time during classification via VEBFNN was 0.105 seconds. It was also indicated that MRMR was not a proper criterion to be used for making more effective feature sets in comparison with RA.

**Conclusions:**

This work was accomplished by introducing the most discriminating facial EMG time-domain feature for the recognition of different facial gestures; and suggesting VEBFNN as a promising method in EMG-based facial gesture classification to be used for designing interfaces in human machine interaction systems.

## Introduction

A recent report released by World Health Organization (WHO) and World Bank shows that more than one billion people with disabilities face substantial barriers in their daily lives [1]. In order to help these people, especially the ones with critical disabilities as the result of strokes, neuro-diseases, and muscular dystrophy, human machine interaction (HMI) has been proposed as a promising way to improve the quality of their lives [2]. Controlling assistive devices, such as wheelchairs [3] and prosthetic limbs [4] are instances in this area. Designing such devices requires applying reliable interfaces as a communication channel between humans and machines. Interfaces that rely on facial neuromuscular activities generated from facial gestures have been lately suggested. The goal here is to recognize facial gestures through facial EMG signals and transform them into input commands to control the devices. The most recent approaches are: the extraction of three facial gestures during speech via four recording channels and transforming them to control commands [5]; controlling a hands-free wheelchair using five different facial myosignals [6]; the application of five facial gestures to design and control a virtual crane training system [7]; the enhancement of human computer interaction by applying six various facial muscle EMG recordings through eight superficial sensors [8]; the use of EMG and visual based HMI to control an intelligent wheelchair [9]; and controlling an electric wheelchair applying six surface facial EMGs [10]. The reliability and flexibility of these systems directly depends on the numbers of classes (gestures), and the methods used for analyzing facial gestures EMGs.

EMG signals are grouped as stochastic and non-stationary and their analysis is too complex [11]; thus, much investigation is needed. Noise reduction, conditioning, smoothing, data windowing, segmentation, feature extraction, dimension reduction and classification are the common stages of recognizing different EMG patterns. Facial gestures recognition ratio mainly depends on the effectiveness of the EMG feature and classification algorithms which are the focus of this paper.

In order to discriminate different muscle movements (gestures), the most prominent parts of the EMGs (features) that represent the characteristics with enough information for classification should be extracted. Various types of features, such as time-domain, autoregressive coefficients, cepstral coefficients, and wavelet coefficients have been applied to classify of upper limb EMG signals [12]. Other types of EMG features have been used in different applications [13-15]. According to previous studies on facial EMG signals, there are some restrictions when analyzing them through their spectrums. This is because of the similarity of facial EMGs frequency components; therefore, they cannot be processed either by frequency-domain or time-frequency distribution algorithms to classify facial gestures [16,17]. These methods can be applied only during muscle fatigue and for inferring changes in motor unit recruitment investigations [18]. More appropriate characteristics of facial EMGs are time-domain ones because of being easy to compute, working based on signal amplitudes, and possessing high stability for EMG pattern recognition [16,19]. There are several methods of time-domain feature extraction; however, to achieve better results, the feature must contain enough information to represent the significant properties of the signal and it must be simple enough for fast training and classification. Extracted features must be trained and classified into distinguishing categories. Hence, a suitable classifier must be considered to provide a fast process and accurate results. Table 1 reviews the related studies of EMG-based facial gesture recognition systems. In these studies, the number of classes and recording channels varied and different facial gestures were considered. As can be seen from the table, only a few methods were investigated for feature extraction and classification. Since this field of study is still in its primary stage, it needs much more investigation.

**Table 1:** Related studies on facial gesture recognition

**Reference**	**Classes**	**Channels**	**Feature(s)**	**Classifier(s)**	**Result(s)**	**Application**
[6]	5	3	MAV	SVM	89.75-100%	Control a virtual robotic wheelchair
[7]	5	3	RMS	SFCM	93.2%	Control a virtual interactive tower crane
[8]	6	8	AV	GM	92%	Recognition system
[10]	6	2	-	Thresholding	-	Electric Wheelchair Control System
[16]	8	3	RMS	SVM, FCM	80.4%, 91.8%	Recognition system
[20]	3	3	Mean, SD, RMS, PSD	Minimum distance	94.44%	Recognition system
[21]	4	-	MAD,SD, VAR	KNN, SVM, MLP	61%, 60.7%, 56.19%	Man–machine interface
[22]	5	2	RMS	FCM	90.8%	Recognition system
[23]	10	3	RMS	FCM	90.41%	Multipurpose recognition system for HMI
[24]	8	3	RMS	ANFIS+SFCM	93.04%	Recognition system for HMI

-: Neither used nor mentioned in the references.

Since there is not much work reported on facial EMG analysis, this paper considers the same setup used in [23] to investigate more on the impact of different facial EMG features on the classification of facial gestures. Therefore, characteristics of ten facial gestures EMGs were explored by extracting ten different time-domain features. The relationship between these features was examined by means of Mutual Information (MI) measure. Moreover, MRMR and RA were employed to select and rank the features for the purpose of constructing feature combinations.

Classification of features through a fast, reliable and accurate algorithm was another objective of this paper. Accordingly, a VEBFNN was applied to classify the single/multi features and evaluate their effectiveness in order to find the most discriminative one based on the recognition performance and the training time. Furthermore, the efficiency and robustness of this classifier was inspected for facial myoelectric signal classification through being assessed and compared with the conventional SVM and multilayer perceptron neural network (MLPNN) methods.

The rest of this paper is organized as follows. The next section describes all the materials needed to record facial EMGs. Then, the methodology of analyzing the EMG signals is explained. Subsequently, experimental results including statistical analysis and detailed discussions are stated. Finally, a brief summary and recommendations for future work are presented in last section.

## Methods and materials

**Figure 1:**
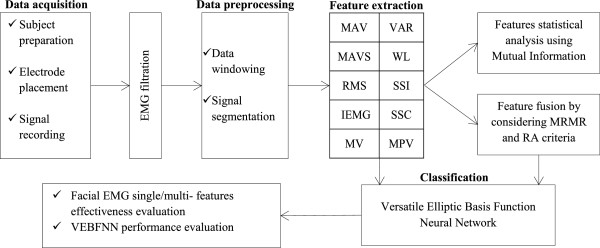
System block diagram of current study.

The procedure of the current study was divided into several steps as demonstrated in Figure 1. The first step consisted of subject preparation, electrode placement, system setup and EMG signals acquisition. Then, all recorded signals were conditioned and filtered prior to processing. Data windowing and segmentation methods were applied in the preprocessing step. Afterwards, ten different types of time-domain features were extracted from all EMG signals. Subsequently, features correlation was analyzed through MI measures. And feature combinations were constructed by considering two criteria MRMR and RA. In order to train and classify the features a very fast VEBFNN was used. This algorithm was employed for the first time to classify EMG signals. Finally, experimental results were discussed in order to evaluate the effectiveness of each feature/combination to find the most discriminative and accurate one that could deliver the highest performance in terms of facial gesture recognition and computational load. Moreover, the efficiency of VEBFNN was assessed and compared with two other popular supervised classifiers, SVM and MLPNN.

### Facial EMG acquisition

#### Subject preparation and electrode placement

**Figure 2:**
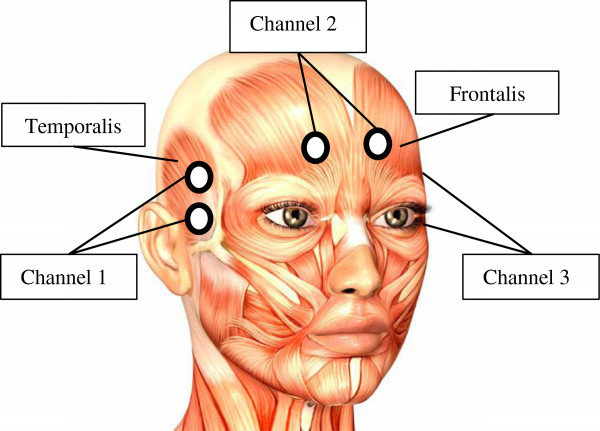
Electrode positions and muscles involved in considered facial gestures.

EMGs are known to be one of the most contaminated signals with a low signal to noise ratio [11]. To achieve clear EMGs, some precautions were considered before signal recording. The subject’s skin was cleaned by means of alcohol pads to remove any dust or sweat in order to reduce the fat layer. In addition, to obtain better signals with higher amplitudes, the electrodes were placed on the right sites [25]. EMGs were recorded through three channels via three pairs of surface rounded pre-gelled Ag/AgCl electrodes. The first and third channels were placed on left and right temporalis muscles and the second channel was positioned on frontalis muscle above the eyebrows (Figure 2). These electrodes were formed in a bipolar configuration (2 cm inter-electrodes distance) on the EMG recording areas to reduce any common noise between them. Another electrode was placed on the boney part of the left wrist to eliminate motion artifacts.

#### System setup and data acquisition

The protocol of this experiment was approved by the Universiti Teknologi Malaysia Human Ethics Research Committee. In the present experiment, facial EMGs were captured via BioRadio 150 (Clevemed) and the signals were recorded at the rate of ~1000 Hz sampling frequency. Through the activation of filters with a low cut-off frequency 0.1 Hz and a notch filter of 50 Hz, unwanted artifacts from user movements and power line inference noises were removed by the device software itself.

Ten mentally and physically healthy volunteers including five male and five female between the ages of 26 and 41 were chosen for this work. Before recording the data, all participants were trained to make facial gestures. The gestures considered for this study were: smiling with both sides of the mouth, smiling with left side of the mouth, smiling with right side of the mouth, opening the mouth (saying ‘a’ in the word apple), clenching the molars, gesturing ‘notch’ by raising the eyebrows, frowning, closing both eyes, closing the right eye and closing the left eye. The subjects were asked to perform each facial gesture five times for two seconds (active signal), and with 5 seconds rest between to eliminate the effect of muscle fatigue. Since the only useful part of a signal for discriminating and recognizing different facial gestures is the active one, only 10 seconds (5×2sec) was considered for the processing of each gesture. Moreover, signals were recorded by the three channels synchronically resulting in a three dimensional data set (3×10 sec) for each gesture. Therefore, ten sets of 3×10 sec active signals were obtained from each subject who performed ten gestures.

### EMG filtration and conditioning

To envelope the most significant spectrum of signals, they were passed through a band-pass filter in the range of 30–450 Hz [7].

### Data windowing and segmentation

Due to the huge amount of data available for processing, the most essential characteristics of facial EMGs (features) should be extracted and considered for further processing. Prior to the feature extraction, filtered signals were segmented into non-overlapped windows with 256 msec length [26]. Since there was a signal of 10000 msec in each channel; 39 portions (10000÷256≈39) were obtained and prepared for feature extraction.

### Feature extraction

Feature extraction is an essential step during EMG processing which has direct effect on final system performance. Good features should highlight the most important properties and characteristics of the facial EMG signal and they should have low computational cost to be used in real-time applications. As mentioned earlier, a number of different features with various complexity and efficiency were suggested and used for EMG signals. In this paper, the ten types of time-domain features extracted from segmented EMGs were Mean Absolute Value Slope (MAVS), Simple Square Integral (SSI), Sign Slope Changes (SSC), Mean Value (MV), Mean Peak Value (MPV), IEMG, WL, MAV, RMS and VAR. The mathematical definition as well as description of these features is provided in Table 2. Since the EMGs were segmented into 39 portions, for each gesture in each channel 39 features were extracted. By considering three channels, a three dimensional feature vector containing 390 features (for 10 gestures) was achieved for each subject using each method.

**Table 2:** Time-Domain features considered in this study

**Feature**	**Equation**	**Description**
MAV	$\mathrm{MA}V_{k}=\frac{1}{N}\sum_{i=1}^{N}\left|x_{i}\right|$	It adds the absolute value of all the values in a segment divided by the length of the segment.
MAVS	$\mathrm{MAV}S_{k}=\mathrm{MA}V_{k+1}-\mathrm{MA}V_{k}$	It estimates the difference between the mean absolute values of the adjacent segments k + 1 and k.
RMS	$\mathrm{RM}S_{k}=\sqrt{\frac{1}{N}\sum_{i=1}^{N}x_{i}{}^{2}}$	It is modeled as amplitude modulated Gaussian random process whose RMS is related to the constant force and non-fatiguing contraction.
VAR	$\mathrm{VA}R_{k}=\frac{1}{N}\sum_{i=1}^{N}{\left(x_{i}-\bar{x}\right)}^{2}$	It is a measure of how far the numbers in each segment lie from the mean.
WL	$WL_{k}=\sum_{i=1}^{N-1}\left|x_{i+1}-x_{i}\right|$	It is the cumulative length of the waveform over the segment. The resultant values indicate a measure of waveform amplitude, frequency and duration.
IEMG	$\mathrm{IEM}G_{k}=\sum_{i=1}^{N}\left|x_{i}\right|$	It calculates the summation of the absolute values of EMG signals (Signal Power estimator).
SSC	$\left\{x_{i}>x_{i-1}\&x_{i}>x_{i+1}\right\}$ and $\left|x_{i}-x_{i+1}\right|\geq \epsilon$	Given three consecutive samples x_i-1_, x_i_ and x_i+1_, the slope sign change is incremented if the equation is satisfied. A Threshold *ϵ* = 0.02
MV	$\bar{x}=\frac{1}{N}\sum_{i=1}^{N}x_{i}$	It represents the EMG potential from any shift in values of the mean.
SSI	$\mathrm{SS}I_{k}=\sum_{i=1}^{N}\left(\left|x_{i}^{2}\right|\right)$	It determines the energy of EMGs in each segment.
MPV	*x*_*k*_ = max |*x*_*i*_|	It is used to find the maximum absolute peak value of EMGs.

In order to investigate the correlation between the single features, the statistical dependence was measured in form of MI which is a more general measurement than a simple cross-correlation [27]. MI is an entropy type quantity, which provides a measure of the amount of information that one random variable contains about another. It can be thought of as the reduction in uncertainty about one random variable given knowledge of the other. Thus, the more mutual information between two random variables *A* and *B*, the less uncertainty there is in *A* knowing *B* or *B* knowing *A* and zero mutual information means the variables are independent [28]. Given two features *A* and *B*, their MI is computed by

(1) $$\mathrm{MI}\left(A;B\right)=\sum_{b\in B}\sum_{a\in A}p\left(a,b\right)\log\left(\frac{p\left(a,b\right)}{p\left(a\right)p\left(b\right)}\right)$$

where *p*(*a*, *b*) is the joint probability distribution function of A and B, *p*(*a*) and *p*(*b*) are the marginal probability density functions of A and B respectively.

It is indicated that a combination of several single features can achieve better recognition accuracy if the features provide complementary information [29]. In this work, the combinations including two to ten features were constructed by considering two feature selection concepts. In pattern recognition, feature selection aims to identify subsets of data that are relevant and best characterizes the statistical property of a target classification variable, which is normally called Maximum Relevance [30]. These subsets often contain material which is relevant but redundant. Among the common measures between features like similarity or correlation coefficient, MI can represent both relevancy and redundancy [30]. The MRMR technique using MI for feature selection was firstly proposed by Peng et al. [30]. The relevance of a feature set A for the class C is defined by the average value of all MI values between the individual feature *f*_*i*_ and the class C as follows:

(2) $$D\left(A,C\right)=\frac{1}{\left|A\right|}\sum_{f_{i}\in A}\mathrm{MI}\left(f_{i};C\right)$$

And the redundancy of all features in the set A is computed by:

(3) $$R\left(A\right)=\frac{1}{{\left|A\right|}^{2}}\sum_{f_{i},f_{j}\in A}\mathrm{MI}\left(f_{i};f_{j}\right)$$

Then, MRMR can be achieved by $\underset{A}{\max}\left[D\left(A,C\right)-R\left(A\right)\right]$

In addition to MRMR, the single features were also selected and ranked based on their individual power in terms of RA. Accordingly, feature combinations were constructed using the rankings appointed by MRMR as well as RA. As stated earlier, each single feature had 3 dimensions (three channels); so, the dimensions of constructed feature combinations including 2, 3, 4, 5, 6, 7, 8, 9, and 10 features were 6, 9, 12, 15, 18, 21, 24, 27, and 30 respectively. For instance, feature set related to the single feature MPV was [*mpv*_*ch*1_, *mpv*_*ch*2_, *mpv*_*ch*3_]^*T*^ while the feature set including two features MPV and MAV was [*mpv*_*ch*1_, *mpv*_*ch*2_, *mpv*_*ch*3_, *mav*_*ch*1_, *mav*_*ch*2_, *mav*_*ch*3_]^*T*^.

### Data classification

**Figure 3:**
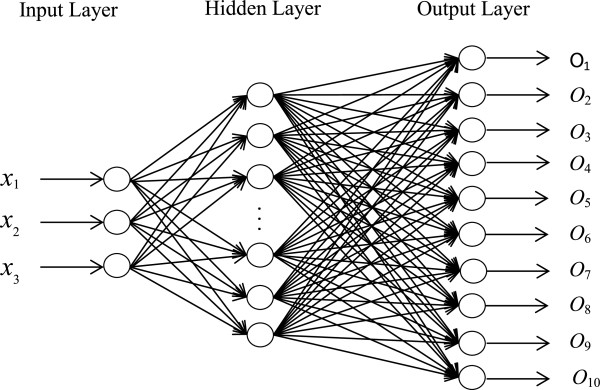
VEBF neural network structure.

To recognize the considered facial gestures, the extracted features must be classified into distinctive classes. A classifier must be able to cope with the factors which remarkably affect the EMG patterns over time such as intrinsic variation of EMG signals, electrode positions, sweat and fatigue. More significantly, a proper classifier has to classify the novel patterns during the online training accurately with very low computational cost to meet real-time processing constraints as the major prerequisite of HMI systems. It was reported that the neural network-based classifiers appropriately addressed the above concerns for myoelectric feature classification [31]. In this study, a VEBFNN was employed to classify the facial EMG features. This method was proposed by Saichon Jaiyen and its robustness was verified and validated by various data sets [32]. The main advantage of this supervised network is that it can learn data sets accurately in only one epoch, and discard datum after passing through which makes it powerful to train the incoming patterns during online training. As reported, this training procedure is very fast in comparison to the traditional neural networks such as MLPNN, and it needs only a small amount of memory [32]. This algorithm also aimed to evaluate the effectiveness of each facial EMG feature on the system performance. The structure of this network depicted in Figure 3 is the same as RBF neural network, which consists of three layers. In the input layer, the number of neurons was equal to the dimension of feature vector, which was three in this study: *x*_*i*_, *i* = 1, 2, 3. The hidden layer, where the number of neurons was not defined in advance since they were formed during the training procedure, was divided into ten sub-hidden layers (number of classes in the training data). The number of neurons in the output layer was also the same as the number of classes in the training data set (ten neurons).

The basis function of neurons in the hidden layer is hyperellipsoid and the output of the *k*th neuron in the hidden layer for each given input *X* = [*x*_1_, *x*_2_, *x*_3_]^*T*^ is calculated by the following equation:

(4) $$\;\psi_{k}\left(X\right)=\sum_{i=1}^{3}\frac{{\left({\left(X-C\right)}^{T}u_{i}\right)}^{2}}{a_{i}^{2}}-1$$

This equation shows a *3*-dimensional hyperellipsoid which is centered at *C* = [*c*_1_, *c*_2_, *c*_3_]^*T*^ and rotated along with orthonormal basis {*u*_1_, *u*_2_, *u*_3_} that enables the neuron to cover neighbor data without translation or any change of size. The width of this hyperellipsoid along each axis is *a*_*i*_, *i* = 1, 2, 3.

**Figure 4:**
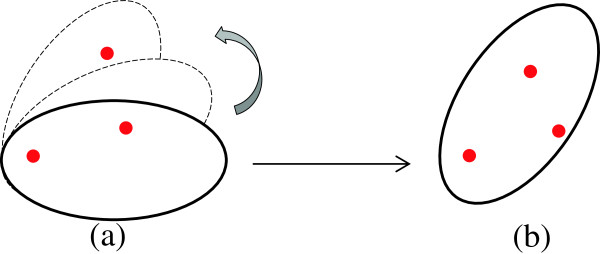
**Data coverage by orthonormal basis rotation. (a)** The attempt of neuron to adjust itself to cover the new data. **(b)** The final position of neuron after new data coverage.

Since the input feature vectors for each sample are in ℜ^3^, the coordinates corresponding to these vectors are standard orthogonal basis [1, 0, 0]^*T*^, [0, 1, 0]^*T*^, and [0, 0, 1]^*T*^. Therefore, component *x*_*i*_ of each input vector *X* with respect to the new axes is computed by *x*_*i*_ = *X*^*T*^*u*_*i*_. The rotation along orthogonal basis vectors enables the neurons to cover all nearby data without increasing the radius. Figure 4(a) shows how the VEBF neuron is trying to adjust itself to cover the new data; finally, the neuron locates as in Figure 4(b).

As mentioned earlier, a feature set with the size of 3×390 (3 is the number of channels) was obtained in the feature extraction step for each subject using each of the different methods. For the purpose of classification, each dataset was shuffled and then divided into 300×3 and 90×3 data features for training and testing stages respectively.

The orthonormal basis was computed through the eigenvectors of the covariance matrix. Since the training data was introduced to the network one by one, the mean vector and covariance matrix were computed recursively. For *N* (300 for each feature set) samples *X* = {*x*_1_, *x*_2_, …, *x*_*N*_} in which *x*_*j*_ = ℜ^3^, *j* = 1, …, *N* the mean vector is calculated by:

(5) $$\mu_{\mathrm{new}}=\frac{N}{N+1}\mu_{\mathrm{old}}+\frac{X_{N+1}}{N+1}$$

where *μ*_*old*_ is the mean vector of the data set *X* and *X*_*N*+1_ is the new data vector added into the data set *X*.

Then the covariance matrix was computed as follows:

(6) $$\tau_{\mathrm{new}}=\frac{N}{N+1}\tau_{\mathrm{old}}+\theta$$

(7) $$\theta =\frac{\left(X_{N+1}X_{N+1}{}^{T}\right)}{N+1}-\mu_{\mathrm{new}}\mu_{\mathrm{new}}{}^{T}+\mu_{\mathrm{old}}\mu_{\mathrm{old}}{}^{T}-\frac{\mu_{\mathrm{old}}\mu_{\mathrm{old}}{}^{T}}{N+1}$$

To find the orthonormal basis for the VEBF, the concept of principal component analysis was considered. Eigenvalues {*λ*_1_, *λ*_2_, *λ*_3_} and the corresponding eigenvectors {*u*_1_, *u*_2_, *u*_3_} were computed from the achieved covariance matrix. Then, the set of eigenvectors, which are orthogonal, form the orthonormal basis. The training procedure is represented in the following.

#### Training procedure

Consider that *X* = {(*x*_*j*_, *t*_*j*_)|1 ≤ *j* ≤ *N*} is a set of *N*=300 training data where *x*_*j*_ is a feature vector (*x*_*j*_ ε ℜ^3^) and *t*_*j*_ is its target. Let *Ω* = {*Ω*_*k*_|1 ≤ *k* ≤ *m*} be a set of *m* neurons. Each neuron has five parameters *Ω*_*k*_ = (*C*^*k*^, *S*^*k*^, *N*_*k*_, *A*_*k*_, *d*_*k*_) where *C*^*k*^ is the center of the *k*th neuron , *S*^*k*^ is the covariance matrix of the *k*th neuron, *N*_*k*_ is the number of data corresponding to *k*th neuron, *A*_*k*_ is the width vector of the *k*th neuron, and *d*_*k*_ is the class label of the *k*th neuron. The whole training procedure can be summarized in the following six steps:

1) The width vector was initialized. Since three dimension feature vectors were used in the current study, a sphere with a radius of 0.5 was considered for simplicity; *A*_0_ = [0.5, 0.5, 0.5]^*T*^.

2) The network was fed with training data set (*x*_*j*_, *t*_*j*_). When no neuron was in the network (*K*=0), *K*=*K*+1 and a new neuron *Ω*_*k*_ was shaped with the following parameters: *C*_*old*_^*k*^ = *x*_*j*_, *S*_*old*_^*k*^ = 0, *N*_*k*_ = 1, *d*_*k*_ = *t*_*j*_, *A*_*k*_ = *A*_0_; then the trained data was discarded. If *K*≠0, the nearest neuron in the hidden layer *Ω*_*k*_ ∈ *Ω* was found such that *d*_*k*_ = *t*_*j*_ and *k* = arg min_*l*_ (‖*x*_*j*_ − *C*^(*l*)^‖), *l* = 1, 2,…,*K*; then, their mean vector and covariance matrix were updated.

3) The orthonormal basis for *Ω*_*k*_ was calculated.

4) The output of *k*th neuron was computed by

(8) $$\psi_{k}\left(X_{j}\right)=\sum_{i=1}^{n}\frac{{\left({\left(X_{j}-C_{\mathrm{new}}^{k}\right)}^{T}u_{i}\right)}^{2}}{{\left(a_{i}^{k}\right)}^{2}}-1$$

If *ψ*_*k*_(*X*_*j*_) ≤ 0, then the neuron covered the data so the temporary parameters were set to its fixed parameters. Otherwise, if *ψ*_*k*_(*X*_*j*_) > 0, then a new neuron was created.

5) Since new neurons can be automatically added to the network and these neurons could be very close together, a merging strategy was considered to avoid growth of the network to the maximum structure (one neuron for each data). The details of this strategy are explained in [32].

6) If there was any more training data, the algorithm was repeated from Step 2; otherwise, the procedure was finished.

## Results and discussion

This section discusses the results of several experiments conducted during the course of this study. First, the classification and recognition accuracy, obtained by training and testing data, achieved by VEBFNN for each feature over all subjects were presented. The impact of each feature on the performance of the recognition system was investigated and compared with others. The computational load consumed during the training stage while using each feature was examined. The effect of each feature on the recognition of each facial gesture was explored. The sensitivity and stability of single features with high discrimination ratios over all subjects were compared. The performances achieved by the most accurate and the one with the lowest level of accuracy were visualized in confusion matrices. Statistical relationships between the considered EMG features were investigated through MI measures. The feature combinations, constructed based on the selected features by MRMR and RA, were examined in terms of recognition accuracy and training time. In the last experiment, the efficiency and reliability of the VEBFNN algorithm was validated by being compared with two conventional classifiers SVM and MLPNN.

### Classification and recognition accuracy

Table 3 presents the classification and the recognition accuracy obtained by VEBFNN for all features and participants. As can be seen, VEBFNN was trained well by different features since the average classification accuracy over all subjects for each feature was above 90%. The maximum degree of accuracy was achieved by MAV (98.5%). On the other hand, the results obtained from the testing stage showed that the ability of VEBFNN for facial gesture recognition varied depending on the type of features used. For instance, notwithstanding that WL features were trained 92.8%; their average recognition accuracy was only 24.5%. The maximum (Test) and minimum (Test) indicated the best and the worst features for each participant based on their achieved test performances. Subjects 1, 2, 3, 6, 7, and 8 reached the maximum recognition performance by utilizing MPV feature; subjects 4, 5, and 9 achieved the highest accuracy by employing IEMG; and subject 10 obtained the best results using RMS feature.

**Table 3:** Classification and recognition accuracy for each subject, Mean value, Standard deviation, and Mean absolute error (%)

**Subject**		**1**	**2**	**3**	**4**	**5**	**6**	**7**	**8**	**9**	**10**	**Mean±SD**	**MAE**
**Feature**													
MAV	Train	98	99.6	98.3	99	98.3	97.3	99	98.3	97.3	99.3	98.5±0.7	1.5
	Test	84.4	85.5	86.7	86.6	85.5	87.6	85.5	85.5	86	86.7	86±0.9	14
MAVS	Train	97.6	96	97	98	98.3	97	97.7	97.6	98	98.4	97.5±0.7	2.5
	Test	83.3	85.6	85.5	83.3	87.7	82.2	85	82.2	84.5	85.5	84.5±1.7	15.5
RMS	Train	98.3	99.3	98.3	97.6	98	96.7	97	95.4	96.7	98.3	97.6±1.1	2.4
	Test	87	84.4	85.5	80	85.6	83.3	86.6	80	83.4	88.9	84.5±2.9	15.5
VAR	Train	100	97.3	99	99.3	98	98.6	97.3	95	100	99	98.3±1.5	1.7
	Test	34	34.4	33.3	33	32	33	32.2	31	35	33	33.1±1.1	66.9
WL	Train	85.3	85	88.3	98	98	97	95	97	85	99	92.8±6	7.2
	Test	22.2	25.5	28	22	26	25.5	24	23.3	27	22	24.5±2.1	75.5
IEMG	Train	99	98	98	99.9	98	97.3	97.3	97.3	96	97.3	97.8±0.9	2.2
	Test	86.6	85.5	82.2	87.7	88.9	86.6	82.2	85.5	86.6	85.5	85.5±2.1	14.5
SSC	Train	93	94	93.6	93	96	95	87	97	98	98	94.5±3.2	5.5
	Test	57	61.1	6	56	59	60	60	58	59	58	58.9±1.5	41.1
MV	Train	86.3	87	94.6	91.3	99.6	98	99	98.6	100	98	95.3±5.2	4.7
	Test	27.7	22.2	25.5	29	33.3	30	30	32.2	32.2	33	30±3.7	70
SSI	Train	95	93.3	95	94.6	94	94	94	91.6	94	93.6	93.9±0.9	6.1
	Test	82.2	85.5	85.6	83.3	80	81.1	80	82.2	83.3	81	82.5±2	17.5
MPV	Train	98	99.6	97.6	96.7	99.3	99.6	97	96.6	95.6	98	97.8±1.4	2.2
	Test	87.7	87.8	87.7	84.4	87.8	87.7	87	87.8	85.5	87.7	87.1±1.1	12.9
Maximum (Test)		MPV	MPV	MPV	IEMG	IEMG	MPV	MPV	MPV	IEMG	RMS	MPV	WL
Minimum (Test)		WL	MV	WL	WL	WL	WL	WL	WL	WL	WL	WL	MPV

**Figure 5:**
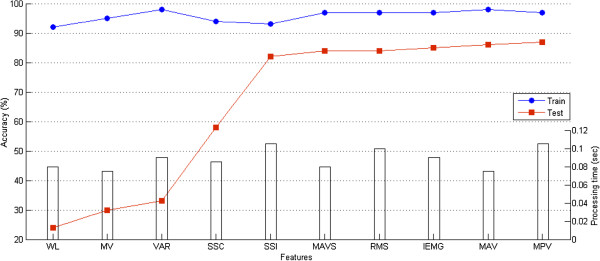
Classification accuracy of training/testing procedures for all features averaged over all subjects and consumed time during training stage.

Figure 5 demonstrates the classification accuracy for all features averaged over all subjects. It shows how different features affect recognition performance. As can be observed, using various features did not result in significant differences in the training performance. In other words, the effectiveness of all features to train VEBFNN was almost similar. On the contrary, the test results determined the real performance and indicated noticeable changes in recognition accuracies by applying diverse features, which delivered different impacts. This figure reported that MAV, MAVS, RMS, IEMG, SSI, and MPV were counted as discriminative and reliable features that contained essential information for the classification of facial states. Amongst them, MPV attained the best performance with the mean recognition accuracy (87.1%) and standard deviation (1.1%) over all subjects whereas WL obtained the lowest result with 24.5% recognition accuracy.

**Figure 6:**
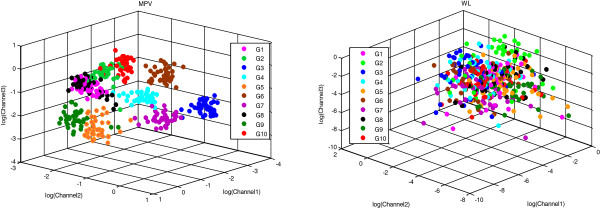
Distribution of MPV and WL features in feature space.

Table 3 also emphasizes the robustness of MPV and the weakness of WL features due to their Mean Absolute Error values over all subjects, which were 12.9% and 75.5% respectively; therefore, they were selected as the most and the least accurate features. Distribution of these two features in the feature space is demonstrated in Figure 6. The classes (gestures) were well-discriminated in MPV features. By contrast, the classes were mixed and could not be recognized from each other in WL features. G1-G10 represent the following facial gestures: opening the mouth (saying ‘a’ in the word apple), clenching the molars, gesturing ‘notch’ by raising the eyebrows, closing both eyes, closing the left eye, closing the right eye, frowning, smiling with both sides of the mouth, smiling with left side of the mouth and smiling with right side of the mouth.

### Computational load

The rate of computation during the training procedure was noted as an important factor in designing the interfaces especially when being used in real-time applications. As can be seen in Figure 5, the consumed training time when using different features was less than a second; explicitly, the maximum time was 0.105 seconds when training MPV and SSI. Overall, this experience proved that VEBFNN was trained very fast using all considered EMG time-domain features which showed the low dependency level of this classifier respect to different features in terms of computational cost. Hence, recognition accuracy was a more reliable metric to compare the capability of features for facial gesture recognition.

### Effectiveness of features on recognition of each facial gesture

In this experiment, we investigated the effectiveness of different features for recognizing each facial gesture using VEBFNN algorithm (Table 4). As can be seen, the best features for the recognition of the facial gestures were as follows: MV for G1; MPV for G2, G3 and G4; MAV, MAVS, IEMG and MPV for G5; MAV and RMS for G6; MAV and MPV for G7; IEMG for G8; MAV and MAVS for G9; and IEMG for G10. According to this table, G3, G5, G7, G9 and G10 were recognized 100% by using different features. Besides, G5 was the most distinguishable gesture since it was accurately recognized with four features whereas G1 was poorly detected considering all features. It is also indicated that MPV provided the highest accuracy for more gestures (5 out of 10) comparing with other features. Therefore, it can be selected as the most proficient feature for single gesture recognition; while, VAR was not effective enough since it resulted in the lowest accuracies for recognizing G2, G6, G8, and G9.

**Table 4:** Recognition accuracy achieved for facial gestures using different features averaged over all subjects (%)

**Gestures**	**G1**	**G2**	**G3**	**G4**	**G5**	**G6**	**G7**	**G8**	**G9**	**G10**
**Features**										
**MAV**	35.5	77.7	88.8	77.7	100	97.7	100	83.3	100	98.8
**MAVS**	31.1	77.7	88.8	77.7	100	94.4	94.4	82.2	100	98.8
**RMS**	25.5	82.2	87.7	86.6	88.8	97.7	96.6	82.2	98.8	98.8
**VAR**	23.3	0	44.4	45.5	55.5	22.2	72.2	14.4	11.1	44.4
**WL**	11.1	32.2	34.4	12.2	11.1	31.1	43.3	24.4	12.2	32.2
**IEMG**	35.5	77.7	88.8	76.6	100	95.5	96.6	88.8	97.7	100
**SSC**	22.2	86.6	45.5	64.4	34.4	70	88.8	43.3	44.4	88.8
**MV**	40	21.1	11.1	11.1	25.5	52.2	42.2	25.5	31.1	35.5
**SSI**	33.3	85.5	87.7	77.7	98.8	81.1	93.3	78.8	90	97.7
**MPV**	36.6	88.8	100	87.7	100	95.5	100	66.6	97.7	98.8
**Mean**	29.41	62.95	67.72	61.72	71.41	73.74	82.74	58.95	68.3	79.38
**Maximum**	40	88.8	100	87.7	100	97.7	100	88.8	100	100
**Minimum**	11.1	0	11.1	11.1	11.1	22.2	42.2	14.4	11.1	32.2

Table 4 also indicates that by considering a same feature for all facial gestures, G1-G10 led to different classification ratios. This may be caused by various reasons such as differences in the involvement of muscles with minor role in shaping each facial gesture; the signal magnitude of muscles which depends on the number of motor units (muscle fibers + motor neuron) and firing rate; action potential resulting from different muscle movements; signaling source of facial gestures; innervation ratio of muscles [33].

### Analytical comparisons of features over subjects

**Figure 7:**
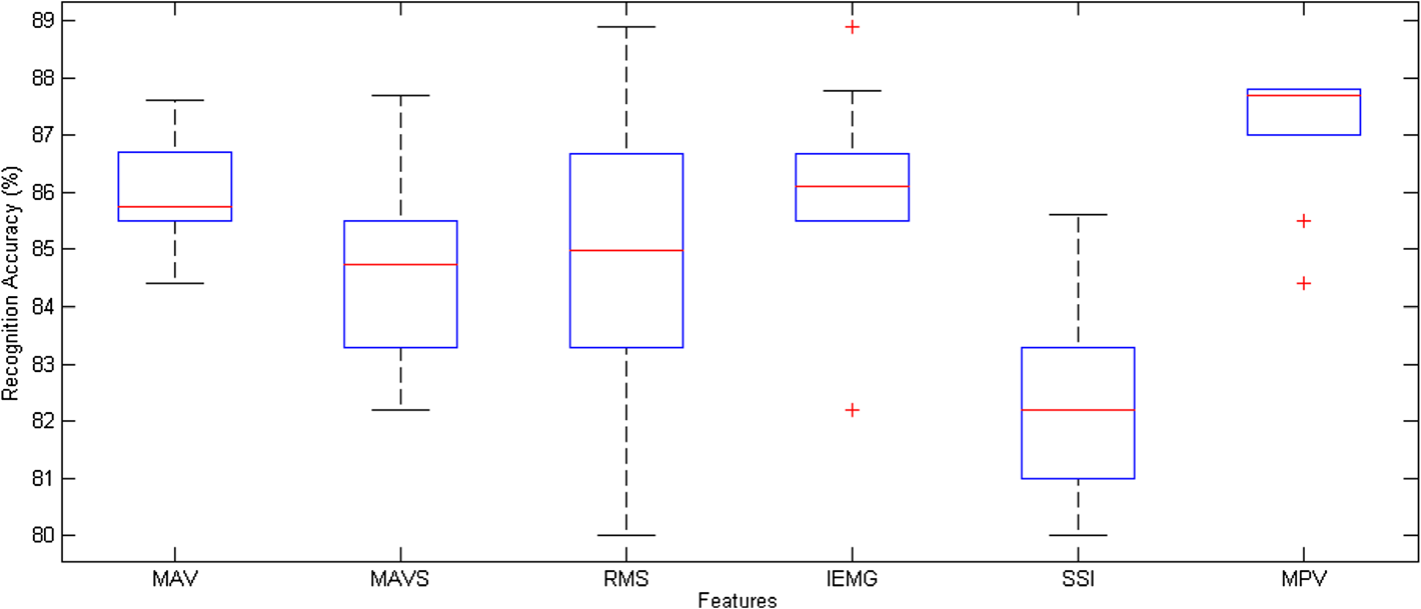
Analytical comparisons of selected features over all subjects.

Further work was carried out to understand the distributional characteristics obtained by VEBFNN over all participants for the features which provided high discrimination ratios: MAV, MAVS, RMS, IEMG, SSI, and MPV. Figure 7 reports that MAV and IEMG had almost the same degree of dispersion since their interquartile were limited in a similar range. MPV was shaped in a short box which meant that all subjects reached close recognition ratios for this feature. In contrast, long spread of accuracies for RMS indicates the high sensitivity of this feature over different subjects. Symmetric boxes for RMS, IEMG, and SSI features point out that the achieved accuracies for different subjects split evenly at the median. The significant point of the figure is the position of MPV median which states that the recognition accuracy exceeded 87% for at least 5 subjects.

### Performance visualization by confusion matrix

The training and testing performances of VEBFNN on the best and the worst single features are visualized as confusion matrices in Tables 5(a) and (b) respectively. These tables illustrate how MPV and WL were classified and misclassified during the training and testing procedures for all facial gestures. As indicated, the significant interaction in Table 5(a) happened between G1 and G8 since in the training stage G1 was 4.3% misclassified in place of G8. This affected the testing stage where just 36.7% of data were recognized correctly. The reason was a similar signaling source for these two gestures. Table 5(b) shows extensive interactions that occurred between all gestures during both training and testing steps which emphasized the weakness of WL for discriminating the facial gesture.

**Table 5:** Confusion matrices averaged over all subjects for (a) MPV and (b) WL features (%)

**(a)**										
***Train***	**G1**	**G2**	**G3**	**G4**	**G5**	**G6**	**G7**	**G8**	**G9**	**G10**
**G1**	95.7	0	0	0	0	0	0	4.3	0	0
**G2**	0	98.3	0	0.3	0.3	0.8	0	0.3	0	0
**G3**	0	0	98.3	0	1.4	0.3	0	0	0	0
**G4**	0	0	0.7	98.3	0	1	0	0	0	0
**G5**	0.7	0	0	0	98	0.3	1	0	0	0
**G6**	0	0	0	0.3	0.3	98.3	0	0.8	0.3	0
**G7**	0	0	0	0	0	0	99.7	0	0	0.3
**G8**	4	0	0	0	0.3	0	0	95.7	0	0
**G9**	0	0.8	0	0.3	0	0.3	0.3	0	98.3	0
**G10**	0	0	0	0	0	0	0.3	1.3	0.7	97.7
***Test***	**G1**	**G2**	**G3**	**G4**	**G5**	**G6**	**G7**	**G8**	**G9**	**G10**
**G1**	36.7	10	0	0	0	0	0	53.3	0	0
**G2**	0	88.9	0	0	0	0	0	11.1	0	0
**G3**	0	0	100	0	0	0	0	0	0	0
**G4**	11.1	0	0	87.8	0	1.1	0	0	0	0
**G5**	0	0	0	0	100	0	0	0	0	0
**G6**	0	0	0	0	0	95.6	0	2.2	2.2	0
**G7**	0	0	0	0	0	0	100	0	0	0
**G8**	14.4	7.8	0	0	0	0	0	66.7	1.1	10
**G9**	0	1.1	0	0	0	1.1	0	0	97.8	0
**G10**	0	0	0	0	0	0	0	1.1	0	98.9
**(b)**										
***Train***	**G1**	**G2**	**G3**	**G4**	**G5**	**G6**	**G7**	**G8**	**G9**	**G10**
**G1**	93	0	0	0.3	1.3	0	1.7	2.7	0	1
**G2**	0	98.7	0	0	0.3	0	0	0.7	0.3	0
**G3**	0	0	96	2.6	0	0	0.7	0.7	0	0
**G4**	2	0	2	90.7	0	0	4.7	0.3	0	0.3
**G5**	0.3	0	0	0.3	94.3	0	0	1.8	3.3	0
**G6**	1.3	0	2.4	2.8	0	90	1.1	1.3	0	1.1
**G7**	1.3	0.3	0	5.7	0	0	91	1	0	0.7
**G8**	2.3	0.7	1.3	1	0.3	3	0.7	86.7	3	1
**G9**	0	0	0.7	0	1	0	1	1	95	1.3
**G10**	3	0	0	1.4	0.3	0.3	1	1	1	92
***Test***	**G1**	**G2**	**G3**	**G4**	**G5**	**G6**	**G7**	**G8**	**G9**	**G10**
**G1**	11.1	11.1	12.2	17.8	0	0	3.3	20	24.5	0
**G2**	0	32.2	32.2	14.4	1.1	4.4	0	0	0	15.7
**G3**	0	0	34.4	34.4	0	0	20	0	11.2	0
**G4**	0	22.3	21.1	12.2	11.1	11.1	10	0	0	12.2
**G5**	22.2	0	12.3	1.1	11.1	8.9	22.2	11.1	11.1	0
**G6**	11.1	0	2.2	0	11.1	31.1	3.4	28.9	0	12.2
**G7**	2.2	0	10	1.1	0	0	43.3	20	11.1	12.3
**G8**	26.7	0	6.7	0	1.1	7.8	1.1	24.4	22.2	10
**G9**	11.1	0	11.1	0	43.3	0	21.2	1.1	12.2	0
**G10**	0	11.1	14.4	3.3	11.1	0	0	16.8	11.1	32.2

### Statistical feature analysis

**Figure 8:**
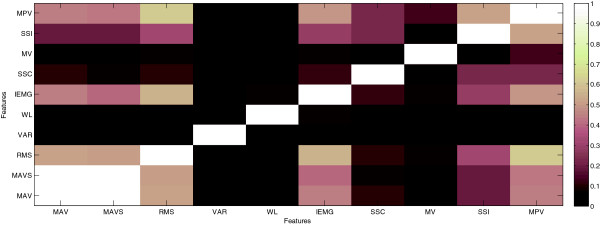
Facial EMG features correlations using Mutual Information measures averaged over all subjects.

In this section, statistical relationships between the single features averaged over all subjects were inspected by means of MI measure (Figure 8). In this figure, brighter pixels stand for higher MI and more relevance between features. The noticeable point is where the MI between MAV and MAVS equaled to 1 which proved that they contained similar characteristics of facial EMGs. The next high degree of relevancy was reported between RMS and MPV, followed by RMS and IEMG whereas SSC and MV had the lowest relationship. Moreover, the very low relevancy of WL with most of the features (MAV, MAVS, RMS, SSC, and MV) denoted either unlike facial EMG information or weakness of this feature in characterizing the EMGs patterns.

### Effectiveness of feature combinations on system performance

This experiment aimed to examine the effectiveness of feature combinations on the system performance. Moreover, the results achieved by these sets were compared with the single feature MPV which was suggested earlier. These combinations were formed based on the rankings shown in Table 6 which were appointed to the single features using MRMR and RA criteria. It can be seen that the feature rankings were different with regard to each criterion. That was due to the fact that MRMR selected the features by considering the relationships among all of them while RA ranked the features with regard to their individual strength in recognizing the facial gestures. According to MRMR, MAV was selected as the best feature whereas based on RA this rank was taken by MPV. Besides, MV reached the second rank via MRMR since this criterion assumed that MV contained complementary information in combinations and might increase the performance; although this feature resulted in too low accuracy as a single feature.

**Table 6:** Feature ranking based on MRMR and RA

**Rank**	**1**	**2**	**3**	**4**	**5**	**6**	**7**	**8**	**9**	**10**
**MRMR**	MAV	MV	MPV	IEMG	SSC	VAR	MAVS	RMS	WL	SSI
**RA**	MPV	MAV	IEMG	RMS	MAVS	SSI	SSC	VAR	MV	WL

**Figure 9:**
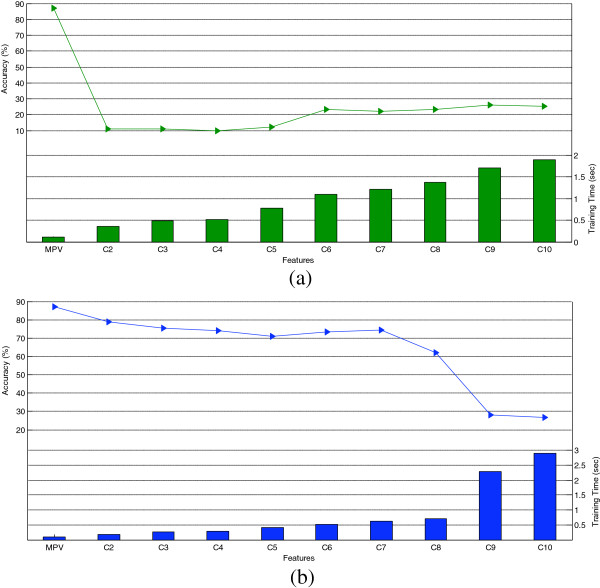
The effect of feature combinations on recognition accuracy and training time by considering (a) MRMR, (b) RA.

In this study, the feature sets including two (C2) to ten (C10) features were constructed as shown in Table 7. The performance of the feature sets formed based on MRMR in terms of recognition accuracy and the consumed training time averaged over all subjects were investigated in Figure 9(a). It can be seen that the recognition performance of all combinations was too low though it was slightly enhanced by increasing the number of features. In addition, it is indicated that the time consumed to train the VEBFNN was raised by applying more features without any considerable improvement in the final system performance. According to Figure 9(b) which demonstrates the performance of the feature combinations formed via RA, once again applying more features generally resulted in lower accuracy and more computational load during the training. Considering C2 in Figure 9(a) and C9 in Figure 9(b), it is observed that the accuracy sharply decreased when MV was added to the combinations. This feature was selected by MRMR as the second one to have the maximum relevancy and the minimum redundancy and it was supposed to improve the system performance by its complementary information. However, MV undesirably impacted the performance since it was very weak in terms of recognition accuracy individually according to the previous findings. On the other hand, the feature sets formed based on RA performed better than those constructed via MRMR which was due to the fact that MV participated in all combinations suggested by the second criterion. Finally, it was proven that all of the feature combinations considered in this study resulted in lower recognition accuracy and consumed more time for training in comparison with the single feature MPV. The main reason was that although some of the single features provided meaningful power for classifying the gestures individually, their combinations not only delivered less discriminative feature sets but also caused more data overlapping between the classes which reduced the classification accuracy.

**Table 7:** Combinations including two to ten features based on MRMR and RA criteria

**Combinations**	**MRMR**	**RA**
C2	MAV,MV	MPV,MAV
C3	MAV,MV,MPV	MPV,MAV,IEMG
C4	MAV,MV,MPV,IEMG	MPV,MAV,IEMG,RMS
C5	MAV,MV,MPV,IEMG,SSC	MPV,MAV,IEMG,RMS,MAVS
C6	MAV,MV,MPV,IEMG,SSC,VAR	MPV,MAV,IEMG,RMS,MAVS,SSI
C7	MAV,MV,MPV,IEMG,SSC,VAR,MAVS	MPV,MAV,IEMG,RMS,MAVS,SSI,SSC
C8	MAV,MV,MPV,IEMG,SSC,VAR,MAVS,RMS	MPV,MAV,IEMG,RMS,MAVS,SSI,SSC,VAR
C9	MAV,MV,MPV,IEMG,SSC,VAR,MAVS,RMS,WL	MPV,MAV,IEMG,RMS,MAVS,SSI,SSC,VAR,MV
C10	MAV,MV,MPV,IEMG,SSC,VAR,MAVS,RMS,WL,SSI	MPV,MAV,IEMG,RMS,MAVS,SSI,SSC,VAR,MV,WL

### VEBFNN efficiency assessment

**Figure 10:**
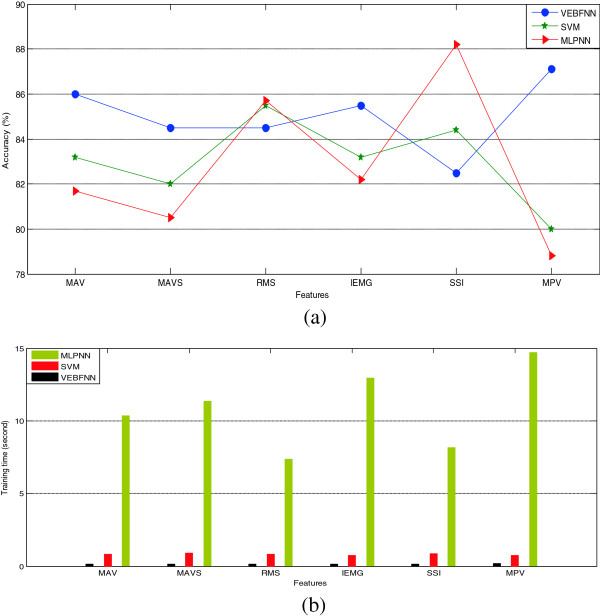
Comparison of VEBFNN, SVM, and MLPNN classifiers over selected features on (a) recognition accuracy and (b) consumed training time.

The following experiment evaluated the robustness of VEBFNN in comparison with SVM and MLPNN. In Figure 10(a), the recognition accuracy achieved by these classifiers was investigated by considering the discriminative single features MAV, MAVS, RMS, IEMG, SSI, and MPV. As can be seen clearly, VEBFNN outperformed the other two classifiers in recognizing the facial gestures when applying MAV, MAVS, IEMG, and MPV features. Besides, all methods delivered almost similar accuracies for the classification of RMS feature. And as observed, MLPNN achieved the highest level of accuracy (88.2%) when classifying SSI. In addition to the above metric, the computational load consumed by these classifiers during the training stage was examined (Figure 10(b)). Comparing all results, it is indicated that MLPNN required too much time for training the features with the minimum of 7.35 seconds for training RMS. As expected, VEBFNN consumed the lowest computational cost since the maximum time was only 0.105 seconds for training MPV. As mentioned before, the purpose of our study was identifying the method which can provide robust performance by considering a reliable trade-off between accuracy and time. Accordingly, although MLP provided the accuracy of 88.2% using SSI; it could not be counted as the best method because the time consumed during training was significantly high, about 8.14 seconds. Therefore, VEBFNN was recommended as the most effective classifier by using MPV feature since it achieved 87.1% accuracy (which is not meaningfully different respect to 88.2% achieved by MLP), and consumed only 0.105 seconds in the training stage.

As stated earlier, facial myoelectric signals have been considered in several studies to design interfaces for HMI systems (Table 1). In [6-8,10,16,20-22,24], the number of employed facial gestures (classes) varied between 3 and 8; whereas, in our study the flexibility of such interface was improved by using ten classes. In terms of feature extraction, a few types of EMG features were focused [6-8,10,16,20-22,24], while in this paper the characteristic of different facial EMG single/multi features were investigated and analyzed comprehensively. For classification of EMG features, this work made use of the accurate and very fast algorithm VEBFNN which was designed and proposed recently; whilst, [6-8,10,16,20-22,24], employed traditional methods. It must be mentioned that, comparing the overall performance of the previous works with the results of this paper was not fair since the number of classes as well as the participants, signal recording protocol and the considered facial gestures were not the same. When comparing with [23] in which a similar setup was considered, it should be noticed that despite the lower accuracy (about 3%) achieved by VEBFNN, this classifier was considerably faster than FCM.

To sum up, due to the fact that real-time myoelectric control requires high levels of accuracy and speed, a trustworthy trade-off must be considered between these two key factors. The main advantage of VEBFNN was that it needed only one epoch to train new data which resulted in very fast training procedure (less than a second). This algorithm was validated using different types of data [32], and its reliability and usefulness was also proved for EMG-based facial gesture recognition in this study. Moreover, in order to find the best recognition performance, various types of facial EMG single features as well as feature combinations were evaluated among which MPV was the most discriminative one.

## Conclusion and future works

In this paper, a reliable facial gesture recognition-based interface to be used in human machine interfacing applications was presented. The effectiveness of ten EMG time-domain single features were explored and compared in order to find the most discriminating. Statistical analysis was carried out by means of MI to reveal the rate of relevancy between the features. The impact of feature combinations, formed based on MRMR and RA criteria, was investigated on system performance and compared with the best single feature. The application of a VEBFNN was proposed and evaluated for the classification of facial gestures EMG signals. The best facial myoelectric feature introduced in this study was MPV which provided the highest discrimination ratio between the facial gestures. Considering this feature, VEBFNN offered a robust recognition performance with 87.1% level of accuracy and very fast training process with only 0.105 seconds. This study clarified that MPV outperformed all the feature combinations constructed through either MRMR or RA criteria in both terms of accuracy and computational cost.

The findings of this study are meant to be practically applied for processing and recognizing the facial gestures EMGs so as to design reliable interfaces for HMI systems. They can also be applied in the fields that require analyzing and classifying EMG signals for other purposes. This technology will be used to control prosthesis and assistive devices that aid the disabled. Designing trustworthy interfaces requires highly efficient methods in terms of accuracy and computational manners. So, in future a more thorough investigation on facial gesture EMGs analysis is recommended and other successful techniques in the field of biomedical signal processing will be examined. Furthermore, as the disabled are intended to benefit from this research, they will be the focus of future studies.

## Abbreviations

EMG: Electromyogram; VEBFNN: Versatile elliptic basis function neural network; WHO: World Health Organization; HMI: Human machine interaction; IEMG: Integrated EMG; MAV: Mean absolute value; MSD: Maximum scatter difference; RMS: Root mean square; PSD: Power spectrum density; AV: Absolute value; MAD: Mean absolute deviation; WL: Wave length; SD: Standard deviation; ZC: Zero crossing; FMN: Frequency mean; FMD: Frequency median; VAR: Variance; SVM: Support Vector machine; MLPNN: Multi-Layer perceptron neural network; K-NN: K-nearest neighbors; FCM: Fuzzy C-means; MAVS: Mean absolute value slope; SSI: Simple square integral; SSC: Sign slope changes; MV: Mean value; MPV: Maximum peak value; SFCM: Subtractive fuzzy C-means; GM: Gaussian model; MI: Mutual information; MRMR: Minimum-redundancy-maximum-relevance; ANFIS: Adaptive Neuro-fuzzy inference system; RBF: Radial basis function; RA: Recognition accuracy.

## Competing interests

The authors declare that they have no competing interests.

## Authors’ contributions

MH conception and design of the study, working on the algorithm design, analysis and interpretation of data, drafting of manuscript, revision of manuscript. S-HS study supervision, contribution in discussion and suggestions, critical revision of the manuscript for important intellectual content, approval of the final version of the manuscript. MA working on the algorithm design, contribution in discussion and suggestions, approval of the final version of the manuscript. AMN contribution in discussion and suggestions, approval of the final version of the manuscript. All authors read and approved the final manuscript.

## Authors’ information

About the Author—Mahyar Hamedi Currently he is a Ph.D. candidate at the Center for Biomedical Engineering, Faculty of Bioscience and Biomedical Engineering, University Technology Malaysia, Johor Bahru, Malaysia. He is IEEE member and his research interests are Biomedical Signal/Image Processing, Human Machine Interaction, Brain Computer Interaction and Neural Engineering. About the Author—Sh-Hussain Salleh Currently, he is a Full Professor, Faculty of Bioscience and Medical Engineering, University Technology Malaysia, director of Center for Biomedical Engineering, IEEE member, IEM member, professional engineer. Project leader: speech recognition and synthesis, heart sound and ECG signal processing, motor imagery signal analysis for brain computer interaction. His research interests are Biomedical Signal Processing, Pattern recognition and neural network. He has published over 100 papers, and he has supervised many Master, PhD and Postdoc students. About the Author—Mehdi Astaraki received the Master of Biomedical Engineering at department of Biomedical Engineering, Science and Research Branch, Islamic Azad University Tehran, Iran. His research interests are Biomedical Signal/Image Processing and neural network. About the Author—Alias Mohd Noor is the dean of Research Alliance with four research centers under his administration one of which is Centre for Biomedical Engineering. He is a Professor in Mechanical Engineering. He is Fellow in Institute of Engineers Malaysia, Professional Engineer in Board of Engineers Malaysia, ASEAN Eng., Int. PE, Asian Chartered Engineers, APEC Eng. He has won several awards from his research products and innovations.
